# Acupuncture for idiopathic pulmonary fibrosis: Protocol for a systematic review

**DOI:** 10.1097/MD.0000000000009114

**Published:** 2017-12-29

**Authors:** Yang Xie, Jia-Jia Wang, Gao-Yang Li, Xuan-Lin Li, Jian-Sheng Li

**Affiliations:** a Department of Respiratory Diseases, the First Affiliated Hospital of Henan University of Chinese Medicine; b Henan Key Laboratory of Chinese Medicine for Respiratory Disease; c Collaborative Innovation Center for Respiratory Disease Diagnosis and Treatment and Chinese Medicine Development of Henan Province, Henan University of Chinese Medicine, Zhengzhou, Henan Province, China.

**Keywords:** acupuncture, idiopathic pulmonary fibrosis, protocol, systematic review

## Abstract

**Background:**

Patients with idiopathic pulmonary fibrosis (IPF) often suffer from progressive worsening of dyspnea and lung function. Although 2 drugs are now available for IPF treatment, many gaps are still to be filled. Evidences suggest that there may be some beneficial effect of acupuncture on pulmonary diseases. This review aims to systematically determine the efficacy and safety of acupuncture for treating IPF.

**Methods:**

The following 8 databases will be searched from their inception to March 20, 2017: PubMed, Embase, Cochrane Central Register of Controlled Trials, Web of Science, Chinese Biomedical Literature Database, China National Knowledge Infrastructure, Chongqing VIP, and Wanfang Data. Randomized controlled trials (RCTs) of acupuncture for IPF without restriction of publication status will be included. Two review authors will independently undertake study selection, extraction of data, and assessment of study quality. Meta-analysis will be conducted after screening of studies if possible. Dichotomous data will be analyzed using odds ratio (OR), and continuous data mean difference (MD). Statistical analysis will be conducted using Review Manager (RevMan) software.

**Results:**

This systematic review will provide a detailed summary of the current evidences related to the efficacy and safety of acupuncture in improving breathlessness, exercise limitation, health status impairment of patients with IPF.

**Conclusion:**

This evidence may be useful to clinicians, patients, and health policy-makers with regard to the use of acupuncture in IPF treatment.

**Ethics and dissemination:**

This systematic review will provide up-to-date information on acupuncture for IPF. This review does not require ethical approval and will be disseminated electronically through a peer-reviewed publication or conference presentations.

**Trial registration number:**

PROSPERO 2017:CRD42017059848.

## Abstract 2

Supplemental Digital Content is available in the text

## 1 Introduction

Idiopathic pulmonary fibrosis (IPF) is a prototype of chronic, progressive, and fibrotic lung disease, characterized by progressive worsening of dyspnea and lung function and is associated with a poor prognosis.^[1,2]^ Incidence of IPF has increased over time in most countries, and incidence range in Europe and North America is conservatively estimated to be 3 to 9 cases per 100,000 per year, yet seems to be lower in Asia and South America.^[3]^

It has been reported that the mortality of IPF ranges from 4 to 10 per 100,000, and is increasing steadily worldwide.^[4]^ The median survival of patients with IPF is only 2 to 3 years.^[5]^ IPF is associated with a large economic and health care burden, especially in those with hospitalizations and exacerbations.^[6–8]^

International evidence-based guidelines have clarified the diagnostic approach and criteria, making large clinical studies possible. Various putative therapies have been identified by clinical trials as harmful, ineffective, or effective in IPF treatment.^[1,2,9–11]^ However, although 2 drugs are now available for IPF treatment, many gaps are still to be filled.^[10–14]^ For example, neither drug improves the symptoms, such as dyspnea and cough. Therefore, therapies for IPF should be global and comprehensive including comorbidities, and nonpharmacological options should be offered as well.^[11]^

Acupuncture, an important part of traditional Chinese medicine, has been used for thousands of years in treating many painful and nonpainful conditions.^[15,16]^ To date, it has become popular and been widely practiced in many countries around the world.^[17]^ In the past 2 decades, acupuncture research has grown markedly in both the proportion of randomized clinical trials (RCTs) and the impact factor of journals.^[17]^ While pain has been a consistently dominant research focus, other topics have gained more attention during this time period as well.^[17]^ Evidences from both clinicians and patients suggest that there may be some beneficial effect of acupuncture on pulmonary diseases.^[16,18]^ There have been some researches that provide evidence for the clinical application of acupuncture for IPF.^[19,20]^ However, the efficacy and safety of acupuncture for IPF have not been reviewed systematically. Thus, evidences need to be collected and analyzed for the evaluation of efficacy and safety of acupuncture for IPF.

## 2 Methods and analysis

The protocol has been registered on PROSPERO 2017 (registration number: CRD42017059848). This protocol adheres to the Preferred Reporting Items for Systematic Reviews and Meta-Analyses Protocols (PRISMA-P) statement guidelines (see Supplementary Table 1, which represents the PRISMA-P checklist).^[21]^ We will document the important protocol amendments in the full review.

### 2.1 Criteria for considering studies for this review

#### 2.1.1 Types of studies

RCTs of either cross-over or parallel group design in which participants were randomly assigned at the individual or cluster level will be included.

#### 2.1.2 Types of participants

Patients with diagnoses of IPF according to ATS/ERS/JRS/ALAT guidelines will be included.^[1]^ But we will exclude RCTs that focused on participants who were mechanically ventilated or who had an acute exacerbation within 4 weeks before commencement of the intervention. There will be no restrictions on age, sex, ethnicity, education, or economic status.

#### 2.1.3 Types of interventions

We will include studies that evaluated any type of invasive acupuncture and needling techniques, such as manual acupuncture, electroacupuncture, auricular acupuncture, or warm acupuncture, but we will exclude studies that used noninvasive techniques such as laser acupuncture, point application, transcutaneous electrical stimulation over acupuncture points, single moxibustion, or acupressure.

Comparisons investigated are:

- Acupuncture versus no treatments;
- Acupuncture versus placebo or sham treatment;
- Acupuncture versus other treatments;
- Acupuncture adjunctive to other treatments versus other treatments alone;
- Acupuncture adjunctive to other treatments versus placebo or sham treatment adjunctive to other treatments.

#### 2.1.4 Types of outcome measures

*Primary outcomes*

- Forced vital capacity (FVC)^[22,23]^
- St. George's Respiratory Questionnaire (SGRQ)^[24]^

*Secondary outcomes*

- Clinical symptoms
- Six-minute walk test/distance (6MWT/6MWD)^[25]^
- Acute exacerbation^[23]^
- The modified Medical Research Council dyspnea scale (mMRC)^[26,27]^
- Effective rate
- Adverse effects

### 2.2 Search methods for identification of studies

#### 2.2.1 Electronic searches

We will search PubMed, Embase, Cochrane Central Register of Controlled Trials (CENTRAL), Web of Science, Chinese Biomedical Literature Database, China National Knowledge Infrastructure, Chongqing VIP, and Wanfang Data from their inception to March 20, 2017. We have developed detailed search strategies for each electronic database without language restrictions to attempt to identify all eligible studies. The search strategy for PubMed is shown in Table 1.

**Table 1:**
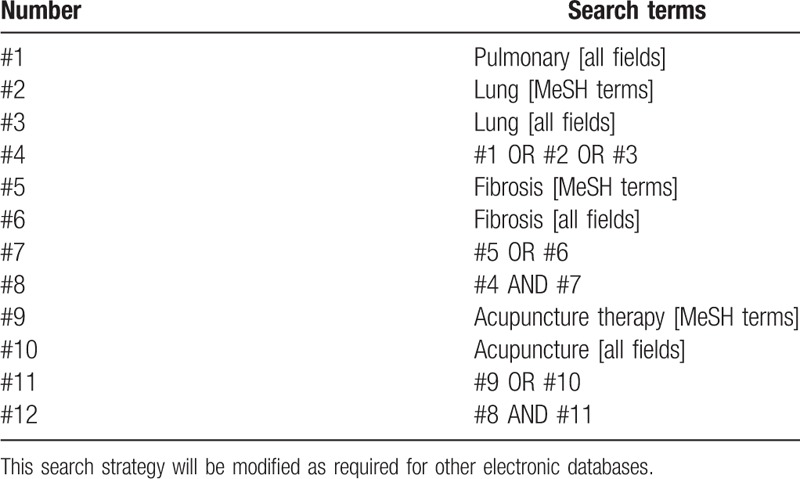
Search strategy for PubMed.

#### 2.2.2 Searching other resources

We will review reference lists of eligible studies to identify further eligible studies.

### 2.3 Data collection and analysis

#### 2.3.1 Selection of studies

**Figure 1:**
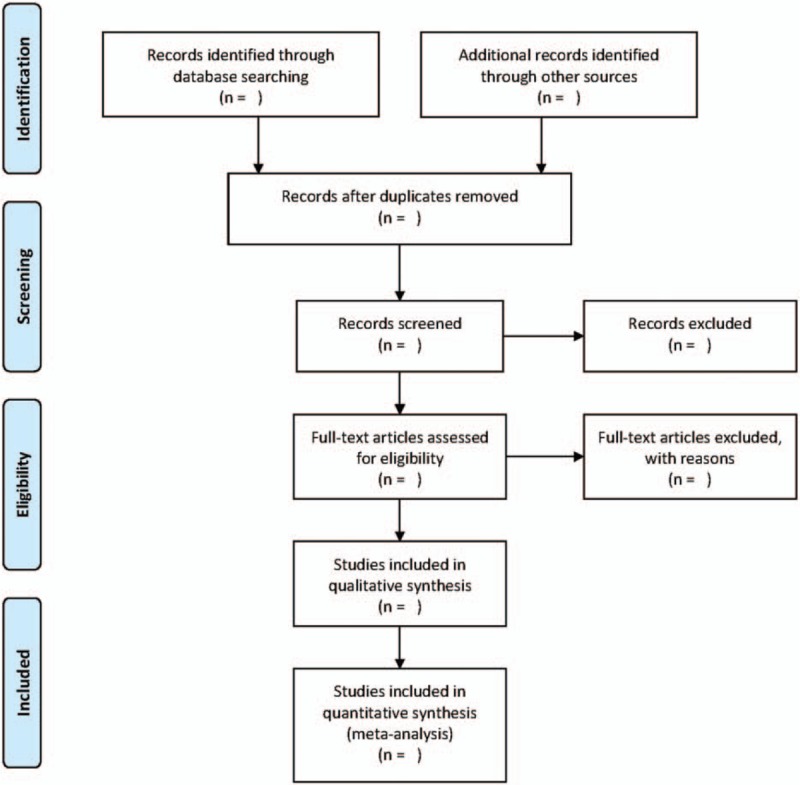
Study flow diagram.

Two review authors (G-YL and X-LL) will independently examine titles and abstracts retrieved from the search and select all potentially eligible studies. Then these full-text articles will be obtained and the same review authors will review them independently against the inclusion criteria used in each study. We will resolve all disagreements by consensus, and a third review author (J-JW) will act as an arbiter when consensus cannot be reached. The review authors will record all studies that do not meet the inclusion criteria and provide the rationale for their exclusion. Details of the selection process will be presented in the PRISMA flow chart (see Fig. 1).^[28]^

#### 2.3.2 Data extraction and management

We will conduct data extraction up to March 2017 and complete a search log that shows the databases searched and the dates of searches. We will complete a data extraction sheet for every study included in the review, involving information on details of authors, year of publication, study design, characteristics of participants, intervention, comparator, and outcomes.

#### 2.3.3 Assessment of risk of bias in included studies

Methodological quality will be independently assessed using the Cochrane tool for assessing risk of bias in RCTs.^[29]^ Two review authors (G-YL and X-LL) will complete the data extraction and score each study, with a third review author (J-JW) acting as an arbiter where differences occur between G-YL and X-LL. We will summarize risk of bias and settle differences in author interpretation of data through discussion.

#### 2.3.4 Measures of treatment effect

We plan to pool data from the outcomes of each study to provide an overall measure of the effect. We will summarize data using odds ratio (OR) with 95% confidence intervals (CI) for dichotomous outcomes, and using mean difference (MD) with 95% CI for continuous outcomes.

#### 2.3.5 Unit of analysis issues

*Cross-over trials* We will use data only from the first half of the trial as a parallel group design.

*Cluster-randomized trials* We will include cluster-randomized trials in the analysis for the current review alongside individually randomized trials. And we will make an adjustment to the sample size in these studies for each intervention based on the method described in the Cochrane Handbook for Systematic Reviews of Interventions.^[29]^

*Multi-armed trials* We will include multiarmed trials in this review. To overcome potential issues due to multiple, correlated comparisons, we will analyze multiarmed trials using methods described in the Cochrane Handbook for Systematic Reviews of Interventions.^[29]^ When feasible, we will combine multiple comparison groups to create 1 relevant intervention group and 1 relevant comparison group.

#### 2.3.6 Dealing with missing data

Included studies with greater than 20% attrition will be considered at high risk of attrition bias.^[30]^ When standard deviations (SDs) of the change of included studies are missing, we will substitute for them the mean SD of other included studies. We will exclude from the analysis studies in which only medians and percentiles are available and there are no means of calculating mean change scores.

#### 2.3.7 Assessment of heterogeneity

We plan to use a *χ*^2^ test to estimate heterogeneity of both the MD and OR. Further analysis can be performed using the I^2^ test. If possible, we will also construct a forest plot for analysis. A random-effect model will be used to interpret the results if heterogeneity is statistically significant, whereas a fixed-effect model will be used if heterogeneity is not statistically significant. We will regard heterogeneity as substantial when I^2^ is greater than 50% or a low *P* value (<.10) is reported for the *χ*^2^ test for heterogeneity.^[31]^

#### 2.3.8 Assessment of reporting biases

When 10 or more studies are included in the meta-analysis, we will investigate reporting biases (such as publication bias) by using funnel plots.

#### 2.3.9 Data synthesis

We will undertake statistical analysis by using Review Manager (RevMan) software.^[32]^ If the data can be combined into a meta-analysis, we will include categorical data only where it can be divided into dichotomous outcomes. If the data cannot be combined in a meta-analysis, we will summarize them in the text and group them by outcome if appropriate. As with cross-over trials, we will consider only the first phase and exclude from the analysis data obtained during the second phase. We will count multiple publications reporting the same group of participants or their subsets as 1 single study.

#### 2.3.10 Subgroup analysis and investigation of heterogeneity

If data permit, we plan to conduct subgroup analyses for different intervention forms (such as acupuncture alone or adjunctive to other treatments) to assess whether the treatment effects are different in different subgroups.

#### 2.3.11 Sensitivity analysis

We plan to conduct sensitivity analyses to assess the impact of methodological quality along with the effect of high attrition rates on the outcomes.

#### 2.3.12 Grading the quality of evidence

We will use the Grading of Recommendations Assessment, Development and Evaluation (GRADE) approach to describe the overall quality of the outcome.^[33]^ The quality of outcomes will be categorized as high, moderate, low, and very low.

## 3 Discussion

To date, no systematic reviews have investigated the efficacy and safety of acupuncture for patients with IPF. This systematic review will provide a detailed summary of the current evidences related to the efficacy and safety of acupuncture in improving breathlessness, exercise limitation, health status impairment, etc., of patients with IPF. This evidence may be useful to clinicians, patients, and health policy-makers with regard to the use of acupuncture in IPF treatment.

## Supplementary Material

Supplemental Digital Content
